# Hyper-palatable foods in elementary school lunches: Availability and contributing factors in a national sample of US public schools

**DOI:** 10.1371/journal.pone.0281448

**Published:** 2023-02-16

**Authors:** Danielle Dilsaver, Kaitlyn Rohde, Lynn Chollet-Hinton, Tera L. Fazzino

**Affiliations:** 1 Department of Psychology, University of Kansas, Lawrence, Kansas, United States of America; 2 Cofrin Logan Center for Addiction Research and Treatment, University of Kansas, Lawrence, Kansas, United States of America; 3 Department of Biostatistics & Data Science, University of Kansas Medical Center, Kansas City, Kansas, United States of America; Pennington Biomedical Research Center, UNITED STATES

## Abstract

**Background:**

School cafeterias are a major point of influence for child nutrition. United States federal legislation requires the presence of important nutrients in school meals. However, legislation overlooks the potential presence of hyper-palatable foods in school lunches, a hypothesized factor that may influence children’s eating behavior and obesity risk. The study sought to 1) quantify the prevalence of hyper-palatable foods (HPF) served in US elementary school lunches; and 2) determine whether food hyper-palatability varied based on school geographic region (East/Central/West), urbanicity (urban/micropolitan/rural), or meal item (entrée/side/fruit or vegetable).

**Methods:**

Lunch menu data (N = 18 menus; N = 1160 total foods) were collected from a sample of six states that represented geographic regions of the United States (Eastern/Central/Western; Northern/Southern) and that had variability in urbanicity (urban, micropolitan, and rural) within each state. A standardized definition from Fazzino et al (2019) was used to identify HPF in lunch menus.

**Results:**

HPF comprised almost half of foods in school lunches (M = 47%; SD = 5%). Compared to fruit/vegetable items, entrées were >23 times more likely to be hyper-palatable and side dishes were >13 times more likely to be hyper-palatable (p values < .001). Geographic region and urbanicity were not significantly associated with food item hyper-palatability (p values >.05). The majority of entrée and side items contained meat/meat alternatives and/or grains and likely aligned with the US federal reimbursable meal components of meat/meat alternatives and/or grains.

**Conclusions and implications:**

HPF comprised almost half of foods offered in elementary school lunches. Entrées and side items were most likely to be hyper-palatable. US school lunches may be a key point of regular exposure to HPF among young children, a risk factor that may elevate child obesity risk. Public policy regulating HPF in school meals may be needed to protect children’s health.

## Background

In the United States (US), the prevalence of childhood obesity has tripled in recent decades [1]. School cafeterias have been identified as an environment-level factor that may substantially impact children’s health [2, 3]. US federal legislation requires that foods provided in school meals contain important nutrients to promote children’s dietary health [4]. However, the legislation overlooks types of foods that may negatively influence children’s dietary intake and obesity risk.

Current federal meal legislation does not regulate types of foods that may be difficult to stop eating, such as hyper-palatable foods, which are a hypothesized mechanism that may elevate child obesity risk [5]. Hyper-palatable foods (HPF) contain combinations of palatability-inducing nutrients (e.g., fat, sugar, sodium, and/or carbohydrates) that yield an artificially rewarding eating experience [6]. HPF may excessively activate brain reward neurocircuitry, while also dampening engagement of physiological satiety mechanisms, which may facilitate overeating [7, 8]. When consumed repeatedly over time, HPF may dysregulate food reinforcement processes, skewing children’s food intake behavior toward HPF and away from foods such as fruits and vegetables [5, 9]. Evidence indicates that there may be a dose response relationship between HPF intake and health consequences [10]; thus early and repeated consumption of HPF among young children is hypothesized to be a key risk factor for excess dietary HPF intake and child obesity risk [5]. Applied to the school food environment, if HPF are regularly available in and consumed in school lunches, school meals may be a key source of regular HPF exposure among young children that could increase their risk for excess dietary intake of HPF and obesity.

It is unknown to what degree HPF are present in school meals; however evidence from the broader US food environment is cause for concern. Using a standardized definition of HPF, we demonstrated that HPF have saturated the US food environment, comprising 69% of foods in the US food supply as of 2018 [11]. Groups of individuals who may be particularly vulnerable to the rewarding effects of HPF, such as infants, also have widespread exposure to HPF and have been found to consume >50% of daily energy from HPF [12]. Given the widespread availability of HPF and documented regular dietary intake of HPF among infants, it stands to reason that HPF may also be regularly available in US public school settings, such as elementary school lunches. Thus, an examination of the availability of HPF in elementary school lunches is needed to understand the degree to which young children may be regularly exposed to HPF, a key factor that may increase children’s risk for excess dietary intake of HPF and childhood obesity [5].

### Purpose

The purpose of the current pilot study was to quantify the availability of HPF in elementary school lunches among a sample of US public schools. The study also evaluated whether the hyper-palatability of foods served in school meals may vary based on school regional location in the US (Eastern, Central, or Western US) and urbanicity (urban, micropolitan, or rural), and type of meal item (entrée, side, or fruit/vegetable).

## Methods

### Procedures

The study collected and analyzed school menu data that were publicly available online from US public elementary schools. Due to the pilot nature of the study, a target sample of six US states was identified to represent several important geographic regions within the US to ensure appropriate sample variability. Specifically, the sample was equally comprised of states that represented Eastern (n = 2 states), Central (n = 2 states), or Western US (n = 2 states) geographical regions of the US, as well as Northern (n = 2 states), Central (n = 2), or Southern (n = 2) US regions. In addition, states included in the sample were required to have ≥2 regions representing each of the following areas of urbanicity: urban/metropolitan, micropolitan, and rural area [13], to provide a wide enough pool of candidate regions from which to sample online menus. Accordingly, the following six US states were selected for inclusion in the study sample: Arizona, Missouri, Oregon, Pennsylvania, Texas, and Virginia. Within each state, three schools from three different school districts were chosen for analysis based on urbanicity: urban/metropolitan, micropolitan, or rural area [13]. Schools that provided detailed nutrient information for menu items and that corresponded with one of the urbanicity codes in each state were included in the study. A total of 18 unique school menus were analyzed across the six states (detailed in S1 Table). All schools were public elementary schools with grades of kindergarten through 5^th^ or 6^th^ grade and served students approximately 5 to 11 years old. Data were collected in September 2019. No human subjects data were used in the study; all data analyzed were publicly available online. Due to the pilot nature of the study, no sample size calculation was conducted.

### Measures

HPF were identified using the standardized definition from Fazzino et al. [6]. The definition specifies combinations of palatability-inducing nutrients at thresholds that are hypothesized to induce hyper-palatability (criteria are detailed below), consistent with evidence indicating a combination of palatability-inducing nutrients may be directly implicated in enhanced palatability [7, 8]. Preliminary evidence indicates that the HPF definition has convergent validity as it identifies foods hypothesized to be hyper-palatable (e.g., fast foods, etc.) and has discriminant validity for (appropriately) not identifying foods hypothesized to not be hyper-palatable (e.g., fresh/raw fruits) [6]. Furthermore, evidence indicates that healthy individuals prefer and select HPF relative to non-HPF [14], likely due to their reinforcing properties. Finally, preliminary evidence has revealed that US infants and young toddlers may consume >50% of daily energy from HPF [12], suggesting that regular HPF intake among US children may begin in infancy.

### Data processing

Menu data were extracted from publicly available lunch menus posted by schools online. Menus provided detailed nutrient data on the foods served in daily school lunches for a period of 3–4 weeks. Food items were generally presented according to the following categories on the menus: main entrées, side dish, and fruits/vegetables. Condiments and toppings were also included on the menus. Menus provided the following nutrient information per food item: total energy, carbohydrates, fat, sodium, sugar, dietary fiber, and serving size. When serving size was missing, the USDA’s Standard Reference Database was used to identify the standard serving size for the corresponding food [15]. An average of 64 total unique food items per school were available for analysis. A total of N = 1160 unique foods items were collected from the lunch menus.

The definition of HPF was applied to all foods in the lunch menus consistent with Fazzino et al. [6]. Percent calories (kcal) from fat, sugar, and carbohydrates (following subtraction of sugar and fiber) were calculated. Percent sodium by food weight was calculated as sodium (in grams) divided by food weight (in grams). Liquids were removed as the HPF definition does not apply to liquids, because they may have different optimum palatability thresholds than solid foods [6]. All foods in the menus that met criteria for one of the following HPF groups were categorized as HPF: 1) fat and sodium (FSOD) (>25% kcal from fat and ≥.30% sodium per serving), 2) fat and sugar (FS) (>20% kcal fat and >20% kcal sugar, and 3) carbohydrate and sodium (CSOD) (>40% kcal fat and ≥.20% sodium) [6].

### Data analysis

Data analysis was conducted using R Statistical Software Version 4.1.2 [16]. The prevalence of HPF in school lunches was calculated by dividing the total number of foods offered in the lunch menus by the total number of HPF in the lunch menus. To characterize the availability of HPF in menus overall at each school, unique, non-duplicate food items were included in analyses. HPF availability was calculated by state, geographic location, urbanicity, and type of meal item. The type of meal item was evaluated as presented on the school lunch menus using the following categories: entrées, side dishes, fruits/vegetables, and condiments/toppings. Given that some of the menu-specified food categories do not directly align with the meal structure that the US federal government uses to legislate school meals, the entrée and side items were descriptively evaluated to determine the degree to which they may corresponded with the following US federal categories of school meals, termed reimbursable meal categories: meat/meat substitute, grains, fruits, and vegetables [17]. Finally, across all menu items, the availability of types of HPF (FSOD, FS, and CSOD foods) was reported for descriptive information.

To test the relative association between school and meal-based factors with food hyper-palatability, a logistic regression model was constructed with geographic region (Eastern, Central, or Western US), urbanicity (urban, micropolitan, and rural), and meal item (entrée, side, fruit/vegetable, or condiment) included as predictor variables and food HPF status (yes/no) as the outcome. Fruits and vegetables were selected as the reference category in the meal item variable because fruits and vegetables when fresh/raw are hypothesized to not be hyper-palatable [6]. Clustering of food items by urbanicity and geographic region was evaluated with a random intercept generalized linear mixed model with a binomial distribution, logit link, and exchangeable covariance structure. However, the random effects for urbanicity and geographic location were close to zero, indicating that within-subject variability was limited and that most of the variability was between subjects. Therefore, final associations were modeled using unconditional logistic regression.

## Results

### Descriptive statistics

HPF availability in school meals was high overall (47%; 546/1160) and was relatively consistent across schools (Table 1). HPF comprised almost half of foods in school meals when examined across school districts (M = 47%; SD = 5%) and states (M = 47%; SD = 5%). Table 2 presents sample foods that met criteria as HPF and foods that were not HPF based on food type. Fat and sodium HPF comprised the greatest percentage of HPF items relative to total items on the school menus (Table 1). Among HPF items, foods with elevated fat and sodium comprised the majority (M = 78%; SD = 6%) of available HPF across districts, followed by foods with elevated carbohydrates and sodium (M = 24%; SD = 10%) and foods with elevated fat and sugar (M = 12%; SD = 5%). Regarding reimbursable meal components categorized by the US government, entrée items were most commonly comprised of meat/meat alternatives (92%; 557/607) and/or grains (78%; 472/607). Side dishes were most commonly comprised of grains (66%; 95/142) or meat/meat alternatives (33%; 47/142). The majority of entrées met criteria as fat and sodium HPF, whereas side items were most commonly carbohydrate and sodium HPF and/or fat and sodium HPF (Table 1).

**Table 1:** Percentage of hyper-palatable foods in school lunch menus.

	HPF Overall			Fat and Sodium HPF	Fat and Sugar HPF	Carbohydrate and Sodium HPF
Variable	n	M (SD)	Range	M (SD)	M (SD)	M (SD)
**Urbanicity**						
Urban	402	49% (5%)	40–55%	37% (9%)	6% (4%)	12% (3%)
Micropolitan	381	47% (5%)	42–56%	37% (7%)	5% (4%)	13% (8%)
Rural	377	45% (7%)	40–55%	37% (8%)	5% (3%)	8% (3%)
**Geographic Location**						
East	336	41% (4%)	36–47%	30% (2%)	4% (3%)	14% (6%)
Central	433	51% (4%)	44–56%	40% (9%)	8% (4%)	9% (5%)
West	391	49% (5%)	43–55%	41% (4%)	4% (3%)	9% (4%)
**Meal Composition**						
Entrée	607	67% (9%)	56–85%	61% (11%)	2% (5%)	10% (10%)
Side	142	51% (16%)	25–86%	25% (11%)	10% (16%)	37% (17%)
Fruit/Vegetable	321	7% (6%)	0–19%	1% (2%)	6% (5%)	1% (2%)
Condiment	90	50% (20%)	0–100%	40% (26%)	14% (12%)	9% (11%)

*Note*. M = mean; SD = standard deviation

**Table 2:** Example foods and hyper-palatability status by food type.

	Food Type			
	Entrée	Side	Fruit/vegetable	Condiment
**HPF**	Cheese pizza; Fish nuggets with whole grain bun; Turkey sandwich with gravy	French fries; Whole wheat bread roll; Blueberry muffin	Baby carrots with ranch dressing; Blueberries with whipped cream; Sweet potato tater tots	Mayonnaise; Beef gravy; Cheese sauce
**Not HPF**	Penne pasta with sauce; Turkey and cheese sandwich; Baked rotini pasta with vegetables	Black beans; Brown rice (unseasoned); Pinto beans	Steamed broccoli; Applesauce; Baby carrots	Ketchup; Salsa; Jam

Note. HPF = hyper-palatable food

### Relative contribution to food item hyper-palatability

Findings of the logistic regression model indicated that compared to fruit/vegetable items, entrées were >23 times more likely to be hyper-palatable (Table 3) and side items were >13 times more likely to be hyper-palatable (Table 3). School geographic region and urbanicity were not significantly associated with the hyper-palatability of meal items when accounting for meal item type (Table 3).

**Table 3:** Factors associated with hyper-palatability of meal items.

Variable	OR	P value	CI
Geographic Region			
East	-	-	-
Central	1.344	.086	0.959, 1.885
West	0.967	.848	0.687, 1.360
Urbanicity			
Urban	-	-	-
Micropolitan	1.020	.904	0.737, 1.413
Rural	0.958	.797	0.690, 1.360
Meal Item			
Fruit/Vegetable	-	-	-
Entrée	23.372	< .0001	15.269, 37.252
Side item	13.981	< .0001	8.370, 24.057
Condiment	10.769	< .0001	6.054, 19.579

*Note*. OR = Odds Ratio; CI = 95% Confidence Interval

## Discussion

School cafeterias are a major point of influence for child nutrition and US federal legislation requires that school lunches contain important nutrients that support children’s dietary health [4]. However, the legislation does not regulate types of foods that may be difficult to stop eating, such as hyper-palatable foods, which are a hypothesized factor that may elevate child obesity risk [5]. The purpose of the current pilot study was to characterize the availability of HPF in a sample of lunch menus from US public elementary schools. The study examined publicly available school lunch menus provided online and employed a standardized definition to identify HPF in all meals [6]. Findings revealed that HPF comprised almost half of menu items offered when examined by school district and across states. In addition, results consistently identified the type of meal item as a key factor in likelihood that a food on the lunch menu was hyper-palatable. Specifically, entrées and side items were substantially more likely to be hyper-palatable compared to fruits/vegetable items. Thus, findings indicate that HPF are highly prevalent among US elementary school lunches, and that children may primarily be exposed to HPF when consuming school lunch entrées and side dishes. School lunches may therefore be a key source of HPF exposure for young children, and may be a risk factor for promoting HPF consumption among elementary school children, which may have detrimental effects on dietary health [5].

Evidence from this pilot study suggests that HPF availability in school meals was high overall and was relatively consistent across schools in sample. Surprisingly, there were few significant differences in HPF availability based on a school’s geographic region or urbanicity, both of which may influence access to healthy foods [18, 19]. The lack of major differences across region or urbanicity may be due to the commonality among school meal vendors nationally; overall limited vendors and processors exist, and their products are distributed nationally [20]. In contrast, meal item type (entrées and side dishes relative to fruits/vegetables) appeared to be most strongly associated with likelihood that a food item was hyper-palatable. The finding is consistent with our analysis of US national food system data that identified many entrées and the majority of grain-based dishes as HPF [6]. Findings from the current study also indicated that entrée and side items were most commonly identified as HPF through the fat and sodium and/or carbohydrate and sodium HPF groups. Thus, elevated sodium appeared to be a common nutrient that contributed to food item hyper-palatability in school lunches. In line with this premise, in the most recent analysis of national school meal data, approximately half of sodium content in school meals was from entrée items [21]. Overall, our findings suggest that despite the nutritional value and quality of US public school lunches, entrée and side items served in school meals were highly likely to be hyper-palatable.

Our study findings also highlighted fruits and vegetables served in school meals as substantially less likely to be hyper-palatable than the other meal item categories. While not entirely surprising, the findings may still be considered useful given that fruits and vegetables can be prepared in a manner that may yield hyper-palatability if they are not served fresh/raw (e.g., preparing broccoli with melted cheese), as we have found previously [6]. Thus, it appears that most fruits and vegetables served in school meals were not modified in a manner that may induce hyper-palatability, which is encouraging. Furthermore, while the prevalence of HPF served in school lunches was high (M = 47%), the prevalence was lower than the prevalence of HPF in the broader US food system (69%) [11]. It may be that the federal legislation requiring the presentence of both fruits and vegetables in all school lunches may be useful in displacing potentially HPF items, given our findings that fruits and vegetables were substantially less likely to be hyper-palatable than other meal items. However, the findings should also be considered within the context of the US federal legislation that requires schools to offer fruits and vegetables; however students are not required to take all offered items [22]. Thus, future work is needed to understand how the presence of HPF in school meals and current legislative requirements may interact to influence a child’s food selection and intake during school meals.

Our study had several limitations. First, the study was a pilot study and thus included a small number of schools. Future work is needed to expand our analysis to a nationally representative sample of schools. In addition, the study collected data that represented a single month period examined cross-sectionally; thus there may be seasonal variation in the menus that was not captured in our study. Collecting data across the academic year was not feasible with our pilot study; however future research should examine HPF availability in school lunches across the academic year. Additionally, the study did not address the availability of sugar sweetened beverages, because the HPF definition does not apply to liquids. However, given the established relationship between sugar sweetened beverage intake and child obesity risk [23], future work should include an evaluation of these beverages. Finally, the study did not measure children’s food intake during the meals; thus future research is needed to characterize the degree to which HPF availability may influence HPF consumption in school meals among elementary school children.

## Conclusions and health implications

Findings indicate that HPF comprise almost half of foods offered in US elementary school lunches, and that students are most likely to consume HPF when eating entrées and side items in school lunches. Overall, evidence indicates elementary school children may be regularly exposed to HPF in school lunches, and that their exposure to HPF is likely through core meal components such as entrées. The high availability of HPF in elementary school meals is a major cause for concern, as repeated consumption of HPF in early childhood may be a key factor in child obesity risk [5]. Future work is needed to characterize the effects of HPF availability in school meals on children’s HPF consumption behavior and obesity risk. If future work confirms the premise that HPF availability in school meals increases HPF intake and obesity risk among elementary school children, findings may suggest that policy legislation may be needed to limit or remove HPF from school meals to better protect children’s health.

## Supporting information

S1 Table Districts selected for inclusion. (DOCX)
