# Supplementary material for: Hyper-palatable foods in elementary school lunches: Availability and contributing factors in a national sample of US public schools
Source: PLoS One. 2023 Feb 16;18(2):e0281448. doi: 10.1371/journal.pone.0281448 (PMC9934344; doi:10.1371/journal.pone.0281448)
Supplement: S1 Table — (DOCX) [file pone.0281448.s001.docx]

**Supplemental Information**

| **Supplemental Table S1. Districts Selected for Inclusion** | | |
| --- | --- | --- |
| **Urbanicity** | **District Name** | **Total Number of Unique Foods** |
| **Eastern US** | | |
| **Pennsylvania** | | |
| Urban | Philadelphia | 73 |
| Micropolitan | Clearfield | 53 |
| Rural | Tioga | 55 |
| **Virginia** | | |
| Urban | Fairfax | 55 |
| Micropolitan | Martinsville | 36 |
| Rural | Carroll | 64 |
| **Central US** | | |
| **Missouri** | | |
| Urban | St. Louis | 73 |
| Micropolitan | Howell | 66 |
| Rural | Shannon | 61 |
| **Texas** | | |
| Urban | Dallas | 81 |
| Micropolitan | Hale | 75 |
| Rural | Lee | 77 |
| **Western US** | | |
| **Arizona** | | |
| Urban | Maricopa | 65 |
| Micropolitan | Gila | 87 |
| Rural | Apache | 75 |
| **Oregon** | | |
| Urban | Clackamas | 55 |
| Micropolitan | Umatilla | 64 |
| Rural | Clastop | 45 |
